# Obesity-associated poor muscle quality: prevalence and association with age, sex, and body mass index

**DOI:** 10.1186/s12891-020-03228-y

**Published:** 2020-03-31

**Authors:** Pedro L. Valenzuela, Nicola A. Maffiuletti, Gabriella Tringali, Alessandra De Col, Alessandro Sartorio

**Affiliations:** 1 Department of Systems Biology, School of Medicine, University of Alcalá, Ctra Barcelona, Km, 33 600 28871 Alcalá de Henares, Spain; 2 Human Performance Lab, Schulthess Clinic, Zurich, Switzerland; 3 Istituto Auxologico Italiano, IRCCS, Experimental Laboratory for Auxo-endocrinological Research, Verbania, Piancavallo (VB) Italy; 4 Istituto Auxologico Italiano, IRCCS, Division of Metabolic Diseases and Auxology , Verbania, Piancavallo (VB) Italy

**Keywords:** Obesity, Muscle function, Skeletal muscle, Disability, Aging

## Abstract

**Background:**

Muscle quality (i.e., the expression of muscle function per unit of muscle mass) has been proposed as a clinically-relevant measure to detect individuals at risk of functional incapacity. Individuals with obesity might be at an increased risk of having poor muscle quality. Thus, we aimed to analyze the prevalence of poor muscle quality in obese individuals, to determine associated variables, and to provide normative values for this population.

**Methods:**

203 individuals with obesity (103 women, age: 18–75 years, body mass index (BMI): 35–64 kg·m^− 2^) participated in this cross-sectional study. Their muscle strength (handgrip dynamometry), muscle power (sit-to-stand test) and muscle mass (bioelectrical impedance analysis) were measured, and muscle quality (strength/power to muscle mass ratio) was compared with reference values obtained in young healthy individuals. Muscle quality was individually categorized as normal, low or poor based on specific muscle strength and power (i.e., strength and power per unit of muscle mass, respectively). Sex and age-specific normative values of specific muscle strength and power were computed for the whole cohort.

**Results:**

Age and being a woman were inversely associated with specific muscle strength, with age being also inversely associated with specific muscle power. A small proportion of participants (6%) presented with an impaired (i.e., low/poor) specific muscle power while most of them (96%) had impaired specific muscle strength. Eventually, 84% of the participants were deemed to have poor muscle quality. Being a woman (odds ratio [OR]: 18.09, 95% confidence intervals [CI]: 4.07–80.38), age (OR: 1.06, 95%CI: 1.03–1.10) and BMI (OR: 1.22, 95%CI: 1.07–1.38) were independently associated with a higher risk of poor muscle quality in adjusted analyses.

**Conclusions:**

These findings show a high prevalence of poor muscle quality among individuals with obesity, with age, sex and BMI being independent predictors.

## Background

Aging is related to a number of structural and functional changes at the neuromuscular level (e.g., muscle mass loss, impaired neuromuscular activation, intramuscular infiltration of non-contractile tissue, fiber type shift) that result in worsened muscle function [1]. This results in impaired muscle quality, that is, worsened strength/power per unit of muscle mass [2]. Research has shown that impaired muscle function is a better predictor of functional limitations and mortality than muscle mass [3, 4]. For instance, individuals with good muscle quality have been reported to be at a lower risk of functional impairment than those with poor muscle quality, whereas an increased risk of functional impairment has been observed in subjects with greater muscle mass but poor muscle quality [5]. These results confirm therefore the importance of muscle quality as a prognostic factor of functional inability and mortality.

Apart from aging, other factors – particularly in relation with lifestyle – can influence muscle quality. Obesity is reaching epidemic proportions, with its prevalence increasing worldwide and having doubled since 1980 [6]. Among several other complications (e.g., cardiovascular diseases), obesity seems to impair skeletal muscle function. Some studies have shown that a higher body mass index (BMI) was associated with greater muscle mass and even with an increased absolute force and power production capacity [7, 8]. However, when normalized to body mass or muscle mass, obese individuals showed impaired muscle function (i.e., decreased muscle quality) [7, 8]. Although obesity and impaired muscle function are considered independent risk factors of morbidity and mortality, the combination of these two conditions has recently been reported to markedly increase the risk of disability [9–11] and mortality [12] compared to the presence of any of these individuals risk factors alone. However, despite the clinical relevance of muscle quality, particularly in individuals with obesity, the prevalence of poor muscle quality in these individuals compared to the general population remains largely unknown.

The main aims of this study were therefore to analyze the prevalence of poor muscle quality and to determine the variables associated with an increased prevalence of poor muscle quality in a heterogeneous group of obese individuals. We also aimed to provide normative values of muscle quality for individuals with obesity of different age ranges, that could potentially be used as a standard for future studies.

## Material and methods

### Experimental design and participants

The present study followed a cross-sectional, observational design, and complies with the STROBE checklist for observational studies. The study took place between April 2014 and February 2015. Participants were recruited through personal interview before a 3-week in-hospital multidisciplinary weight-management program. Inclusion criteria were having obesity of grade II or more (BMI > 35 kg·m^− 2^) and being older than 18 years. There were no particular exclusion criteria, apart from not being able to perform the physical tests. Participants were additionally categorized as young adults/adults (18–44 years) and middle-aged/older adults (45–74 years) following the recommendations of Spirduso et al. [13]. They had the procedures explained by the main investigator and subsequently provided written informed consent. The study was conducted in accordance with the Declaration of Helsinki and was approved by the Institutional Review Board of the Italian Institute of Auxology.

### Measures

#### Muscle strength

Maximum voluntary handgrip strength was measured with a hand dynamometer (Lafayette Instrument, Lafayette, IN) as explained elsewhere [14]. Briefly, participants were instructed to exert as much pressure as possible for at least 4 s. They performed three trials with each hand, which were interspersed with rest periods of 20 s. The maximum score (in kg) for each hand was recorded, and the mean score of the two hands was used for analysis. Participants were allowed to adjust the dynamometer to their hand size at the beginning of the test, and this position was then kept constant for all tests of that given participant. This test has been extensively used for the assessment of muscle strength, and its validity and reliability have been widely confirmed [15].

#### Muscle power

Muscle power was estimated from the sit-to-stand (STS) test as explained elsewhere [16]. Briefly, participants were required to stand-up and sit-down on a standard chair 10 consecutive times as fast as possible, and the time taken was measured with a stopwatch. Muscle power was computed using the following equation:

$$ \mathrm{Muscle}\ \mathrm{power}\ \left(\mathrm{W}\right)=\left(\mathrm{L}-0.4\right)\times \mathrm{body}\ \mathrm{mass}\times \mathrm{g}\times 10/\mathrm{T} $$

where L corresponds to the participant’s limb length (in meters) measured from the greater trochanter of the femur to the malleolus lateralis, 0.4 corresponds to the height of the chair (in meters), g corresponds to the acceleration of gravity (9.8 m·s^− 2^), and T corresponds to the time (in seconds) taken to perform the test. The STS tests has been shown to be reliable in different populations with impaired muscle function, including older adults [17].

#### Muscle mass

Muscle mass was estimated by means of bioelectrical impedance analysis (BIA) using the following equation [18]:

$$ \mathrm{Muscle}\ \mathrm{mass}\ \left(\mathrm{kg}\right)=\left[\left({\mathrm{height}}^2/\mathrm{R}\times 0.401\right)+\left(\mathrm{sex}\times 3.825\right)+\left(\mathrm{age}\times -0.071\right)\right]+5.102 $$

where height is expressed in cm, R corresponds to BIA-provided resistance in ohms, sex corresponds to 1 and 0 for men and women, respectively, and age is expressed in years. Whole-body resistance to an applied current (at 1, 5, 10, 50 and 100 kHz, 0.8 mA) was measured with a tetrapolar device (Human IM, Dietosystem, Milan, Italy). The electrodes were placed on the right wrist and ankle of the participants while lying supine in a bed. Measurements were performed after an overnight fast. Participants were asked not to drink within 4 h before the test, not to consume caffeine or alcohol within 12 h before the test, and to empty their urinary bladder at least 30 min before the test.

#### Muscle quality

**Fig. 1:**
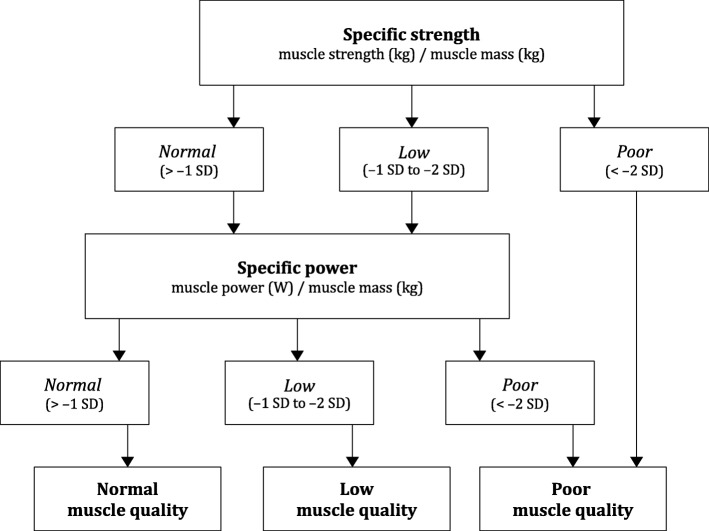
Criteria for the categorization of muscle quality (cut-off values from Barbat-Artigas et al. [2])

Muscle strength and muscle power were expressed relative to muscle mass to provide specific muscle strength (i.e., muscle strength per unit of muscle mass, in kg/kg) and specific muscle power (i.e., muscle power per unit of muscle mass, in W/kg), respectively. The cut-off points proposed by Barbat-Artigas et al. [2] for young healthy individuals were used to categorize participants into having normal, low, or poor specific muscle strength and specific muscle power. Muscle quality (i.e., an overall reflect of muscle function per unit of muscle mass) was categorized as normal, low or poor following the algorithm shown in Fig. 1. We also used the same algorithm to compute sex- and age-specific normative values of specific muscle strength and specific muscle power (and thus to classify muscle function as normal, low or poor) for obese individuals.

### Statistical analysis

Data are shown as mean ± SD (continuous variables) or as numbers and percentages (dichotomous variables). Between-sex and -age group comparisons were performed with Student’s unpaired t-tests and chi-square tests (or Fisher’s exact tests when applicable). Linear regression analyses were conducted to analyze the relationship between specific muscle strength or power and age, sex or BMI. Odds ratios (OR) along with 95% confidence intervals (CI) were computed by means of binary logistic regression to assess the association between these variables and poor muscle quality. Variance inflation factors (VIF) were examined to inspect for multicollinearity and were set at a maximum of 5 (VIF were in all cases < 2.2). Nagelkerke R^2^ values were computed as a measure of the goodness of the fit. Statistical analyses were performed using SPSS (23.0, IBM, Armonk, NY) with a level of significance of *p* <  0.05.

## Results

From a total of 218 initially-enrolled individuals, 15 could not perform the STS test and were therefore excluded. One participant could not perform the STS test but he had previously performed the handgrip test and was deemed to have poor muscle quality. Thus, there was no need to perform the STS test in this individual. Finally, 203 obese individuals (103 women, age: 18–75 years, BMI: 35–64 kg·m^− 2^) met the inclusion criteria and were included in the study. Young adults/adult men were older than women, but no sex-related differences were found for BMI (Table 1). Regardless of age, men presented with a higher muscle mass, absolute muscle strength and power, and specific muscle strength than women. Women presented with a higher specific muscle power than men in the younger but not in the older group.

**Table 1:** Descriptive characteristics of the participants by age group and sex

	Young adults/adults (18–44 years)			Middle-aged/older adults (45–74 years)		
	Men (*n* = 51)	Women (*n* = 41)	p-value	Men (*n* = 49)	Women (*n* = 62)	*p*-value
Age (years)	34.0 ± 8.1	30.1 ± 8.4	**0.026**	54.1 ± 7.7	56.5 ± 7.5	0.098
BMI (kg·m^− 2^)	42.2 ± 4.2	41.6 ± 5.1	0.553	42.2 ± 4.6	43.7 ± 5.9	0.198
Muscle mass (kg)	38.0 ± 4.3	24.5 ± 2.7	**< 0.001**	37.1 ± 4.8	22.7 ± 2.8	**< 0.001**
STS time (s)	16.1 ± 5.5	15.0 ± 4.8	0.304	17.7 ± 5.3	22.4 ± 8.6	**0.001**
Muscle power (W)	418 ± 155	326 ± 116	**< 0.001**	361 ± 125	222 ± 89	**< 0.001**
Specific power (W·kg^− 1^)	11.0 ± 3.9	13.2 ± 5.2	**0.015**	9.7 ± 2.9	9.7 ± 4.1	0.939
Muscle strength (kg)	46.0 ± 10.6	26.5 ± 6.6	**< 0.001**	43.5 ± 8.8	22.0 ± 6.6	**< 0.001**
Specific strength (kg·kg^− 1^)	1.21 ± 0.26	1.07 ± 0.29	**0.028**	1.18 ± 0.22	0.96 ± 0.27	**< 0.001**

Data are mean ± SD. The *p*-values correspond to the comparison between sexes

Abbreviations: *BMI* body mass index, *STS* sit-to-stand

Linear regression analyses showed that a higher specific muscle strength was associated with a younger age and being man, but no association was found with BMI (Table 2). On the other hand, a higher specific muscle power was associated with a younger age, but not with sex or BMI (Table 2).

**Table 2:** Relationship between specific muscle strength or power and age, sex or BMI

	Specific muscle strength β (95% CI)	Specific muscle power β (95% CI)
Age (years)	−0.005 (− 0.008, − 0.002) *p* < 0.001	−0.12 (− 0.15, − 0,08) *p* < 0.001
Sex (women)	−0.19 (− 0.27, − 0.12) *p* < 0.001	0.74 (− 0.44, 1.92) *p* = 0.218
BMI (kg·m^− 2^)	− 0.005 (− 0.13, 0.003) *p* = 0.201	−0.001 (− 0.12, 0.12) *p* = 0.993

Data are shown as unstandardized regression coefficients and 95% confidence intervals (CI)

Categorization of muscle-related variables is shown in Table 3 for the entire cohort and for the two age groups; sex-specific differences are also provided (Additional file 1). Although specific muscle strength was low/poor in the quasi-totality of the group, specific muscle power was categorized as normal in most participants. Consequently, there was a very high prevalence of poor muscle quality among both young adults/adults (75%) and particularly among middle-aged/older adults (92%). A higher prevalence of poor specific muscle strength (94 vs 74% for women and men, respectively, *p* <  0.001) and muscle quality (94 vs 74% for women and men, respectively, p <  0.001) was observed in women compared to men, but no sex-related differences were found for the prevalence of low/poor specific muscle power (Additional file 1). None of the participants were deemed to have low muscle quality.

**Table 3:** Classification of specific muscle strength, specific muscle power, and muscle quality of individuals with obesity, also by age group, compared to healthy individuals

Classification	All subjects (*n* = 203)	Young adults/adults (*n* = 92)	Middle-aged/older adults (*n* = 111)	*p*-value
Specific muscle strength				
Normal	8 (4%)	5 (5%)	3 (3%)	**0.003**
Low	24 (12%)	18 (20%)	6 (5%)	
Poor	171 (84%)	69 (75%)	102 (92%)	
Specific muscle power				
Normal	191 (94%)	89 (97%)	101 (91%)	**0.039**
Low	9 (4%)	2 (3%)	7 (6%)	
Poor	3 (2%)	0 (0%)	3 (3%)	
Muscle quality				
Normal	32 (16%)	23 (25%)	9 (8%)	**0.001**
Low	0 (0%)	0 (0%)	0 (0%)	
Poor	171 (84%)	69 (75%)	102 (92%)	

Data are shown as number of participants and percentage

The *p*-values correspond to the comparison between age groups

Regression analyses with the entire cohort showed that age, BMI and being a woman were independently and positively associated with the prevalence of poor muscle quality (Table 4), with these variables together explaining 32% of the variance in muscle quality (R^2^ = 0.317, all variables contributing significantly to the model).

**Table 4:** Relationship of age, sex and BMI with the odds of having an impaired (i.e., low/poor) muscle quality

	Crude OR (95% CI)	Adjusted OR (95% CI)*
Age (years)	1.05 (1.02, 1.08) ***p*= 0.002**	1.06 (1.03, 1.10) ***p*= 0.001**
Sex (women)	5.68 (2.22, 14.51) ***p*< 0.001**	18.09 (4.07, 80.38) ***p*** **< 0.001**
BMI (kg·m^−2^)	1.16 (1.04, 1.30) ***p*= 0.006**	1.22 (1.07, 1.38) ***p*= 0.003**

Data are shown as odds ratio (OR) together with 95% confidence intervals (CI)

*Adjusted for age, sex, height and body mass index (BMI)

The sex- and age-specific reference values of specific muscle strength and power for obese individuals are shown in Table 5.

**Table 5:** Normative values of specific muscle strength and power for individuals with obesity

	Young adults/adults (18–44 years)		Middle-aged/older adults (45–74 years)	
	Men	Women	Men	Women
Specific muscle strength (kg·kg^−1^)				
Normal	> 0.96	> 0.83	> 0.96	> 0.63
Low	0.70–0.96	0.57–0.83	0.74–0.96	0.32–0.63
Poor	< 0.70	< 0.57	< 0.74	< 0.32
Specific muscle power (W·kg^−1^)				
Normal	> 7.19	> 8.48	> 6.79	> 5.29
Low	3.31–7.19	3.61–8.48	3.90–6.79	1.76–5.29
Poor	< 3.31	< 3.61	< 3.90	< 1.76

## Discussion

The present study analyzed muscle strength, power and quality in a cohort (*n* = 203) of obese men and women with a wide range of ages (18–75 years). Our results show a very high prevalence of poor muscle quality among young adults/adults (75%) and particularly among middle-aged/older adults (92%) compared with reference values obtained in healthy individuals aged 18–30 years. This result was mostly explained by poor specific muscle strength, while the prevalence of poor specific muscle power was surprisingly low in the entire cohort and null in the younger age group. We also observed that age, sex and BMI were independent predictors of poor muscle quality. These results are of major clinical relevance, as both obesity and impaired muscle function are linked to a greater risk of functional limitations [9–11] and mortality [12] than any of these conditions alone.

Although the exact mechanisms underlying obesity-associated impairments in muscle function remain to be elucidated, potential determining factors include metabolic abnormalities (i.e., increased oxidative stress, inflammation and anabolic resistance), a shift towards type I muscle fibers, and muscle fat accumulation [8, 19]. Controversy exists regarding the influence of obesity on muscle fiber type distribution [20]. However, Choi et al. [21] found that, although obese older adults presented with greater muscle mass, they also showed a greater proportion of type I muscle fibers (which are less powerful than type II) and a two-fold greater intramyocellular lipid content (e.g., number of lipid droplets and droplet area) than their non-obese counterparts. Interestingly, the same authors observed an inverse relationship between the number of lipid droplets and single muscle fiber contraction velocity and specific power [21]. Greater levels of both total and visceral body fat have been found to be associated with lower levels of muscle density, with the latter being in turn associated with physical performance impairments in individuals with obesity [22]. Recent research concluded that a greater waist circumference (a marker of visceral adipose tissue) was related to a lower muscle quality in a cohort of overweight and obese adults, but weak associations were found for the rest of the abnormalities characterizing the metabolic syndrome (i.e., blood pressure and levels of glucose, triglycerides, and cholesterol) [23]. Further research is however needed to elucidate the factors that relate obesity to impaired muscle function.

Also noteworthy is the observed association between sex and poor muscle quality. Some authors proposed that sex-specific differences in absolute muscle strength were due to greater muscle size in men [24]. However, in the present study differences remained significant after accounting for the amount of muscle mass. In agreement with our results, previous authors have also reported a higher specific muscle strength in men than in women [25]. Several physiological features have been proposed to explain potential sex-related strength differences, including a greater proportion of type I muscle fibers in women and differences in sex-specific hormones (e.g., testosterone) [26]. Moreover, the high cut-off values set for specific muscle strength in women in the study of Barbat-Artigas – which are surprisingly similar to those of men – could also contribute to a greater prevalence of impaired muscle quality in this population [2].

Our results also show that age was positively associated with the risk of having poor muscle quality independently from BMI, which is in agreement with other studies [4]. Indeed, a significant inverse association was observed between age and both specific muscle strength and power. Aging is associated with a deterioration in neuromuscular structure and function that includes the loss of α-motoneurons and decline in motor unit firing rates and voluntary activation [27, 28]. Age-related structural muscle changes also contribute to muscle quality impairment, as they result for example in intramuscular infiltration of non-contractile tissue (i.e., fat and collagen) that might impair physical performance [29, 30]. For instance, Marcus et al. reported that intramuscular adipose tissue was inversely related to physical performance in older adults [31]. Moreover, aging results in a shift towards a higher proportion of type I muscle fibers, muscle fiber atrophy – especially in type II fibers [32] – as well as in changes in muscle structure (i.e., pennation angle and fascicle length) [33]. In this regard, both ageing and obesity are thought to have similar physiological consequences at the muscle level, but their synergistic effects may potentially exacerbate morbidity and mortality [8].

Previous research has also documented a high prevalence of other markers of low physical fitness in overweight and obese older adults. In the LIFE study, which involved individuals aged ~ 79 years with a mean BMI of 30 kg·m^− 2^, participants had a mean score of 7.4 in the short physical performance battery (SPPB) and almost half of these participants presented a score < 8, which is deemed to be indicative of an impaired physical performance [34]. In the ongoing SPRINTT project, which aims to analyze a similar sample (participants aged ~ 79 years with a mean BMI of 29 kg·m^−^^2^, with > 30% of them having obesity), 80% of the participants presented with a SPPB score < 8 [35]. These findings raise awareness on the importance of implementing multidisciplinary programs including exercise training and, in the case of overweight/obese individuals, dietary interventions to reduce the prevalence of impaired muscle quality in this population. In this regard, the LIFE study showed that physical activity programs can successfully reduce mobility disability among overweight older adults [34]; strategies such as resistance training also have the potential to improve specific muscle power (as measured through the STS test used here) and, consequently, muscle quality [36]. Future research should confirm if such interventions can reduce the prevalence of impaired muscle quality among obese individuals of different ages such as those included in our current study.

It is important to note that, although most participants presented a poor level of specific strength, the prevalence of poor specific muscle power was very low in the entire cohort and even null in the younger age group. The handgrip test is one of the most practical options for the evaluation of muscle strength, and has been shown to provide similar information regarding mortality risk than other more complex measures such as knee extension strength [3]. However, muscle power declines at a greater rate than muscle strength with aging [37], and has been proposed to be a better indicator of functional ability [37]. It has been suggested that, contrary to muscle strength, muscle power might be less dependent on muscle mass and more dependent on other factors such as muscle fiber type or neuromuscular properties (e.g., motor unit behavior) [37]. Thus, it could be hypothesized that, although obese individuals present with an impaired specific strength due to a degeneration of intramuscular factors (e.g., fat infiltration), their neuromuscular properties might be relatively well preserved. It must also be noted that in the current study muscle power was estimated with a test originally proposed for the assessment of older adults [16]. However, the reference values used here for the categorization of specific muscle power were obtained from the young cohort assessed by Barbat-Artigas et al. [2], which also may have influenced our findings. Whether other power tests (e.g., jump ability, isokinetic knee extension) provide the same results remains to be elucidated. Moreover, future research should also analyze if individuals with obesity could present a poorer muscle quality in the upper than in the lower limbs, as the latter might be more ‘trained’ due to the necessity of carrying excess body weight/fat as compared to normo-weight individuals.

Some limitations of this study must be acknowledged. We assessed muscle quality based on the index and cut-off values proposed by Barbat-Artigas et al. [2], and although it was proposed as a method to identify individuals at risk of disability, the values were obtained in healthy individuals aged 18 to 30 years. Future research should provide reference values for healthy and non-obese individuals of different age ranges, including older adults. Also noteworthy is the lack of individuals categorized as having low muscle quality in the present study, which might be reflective of low sensitivity of the methods used. Moreover, the small number of women with normal muscle quality (*n* = 6) and a potential risk of over-adjustment – although multicollinearity was checked – might have contributed to the wide confidence intervals found for sex in regression analyses. As a matter of fact, there is a great heterogeneity in the scientific literature in the methods used to assess muscle quality [38], and therefore the results of our study might have been different with an alternative method. Finally, although we accounted for some possible risk factors, other variables such as the amount of body fat or physical activity were not measured, which could have provided a deeper insight into the mechanisms underlying the obesity-related impairment in muscle function.

## Conclusions

The present study shows a high prevalence (75–92%) of poor muscle quality among individuals with obesity of different ages (18–75 years), which was mainly due to poor specific strength (not power). We also observed that age, BMI and being a woman increased the odds of presenting poor muscle quality independently of other risk factors. These findings highlight the importance of implementing tailored interventions (e.g., resistance training and diet) to reduce the prevalence of obesity-associated poor muscle quality. Finally, we also provided normative values of specific muscle strength and power in individuals with obesity of different ages, which could be useful for future studies that aim to further explore the prevalence and potential treatments for poor muscle quality in this population.

## Supplementary information

**Additional file 1.** Sex-specific classification of specific muscle strength, specific muscle power, and muscle quality of individuals with obesity compared to healthy individuals.

## Data Availability

Data will be made available upon request to the corresponding author (Pedro L. Valenzuela, pedrol.valenzuela@edu.uah.es).

## Abbreviations

BIA: Bioelectrical impedance analysis
BMI: Body mass index
CI: Confidence interval
OR: Odds ratio
SPPB: Short physical performance battery
STS: Sit-to-stand test
VIF: Variance inflation factors
