# Supplementary material for: Obesity-associated poor muscle quality: prevalence and association with age, sex, and body mass index
Source: BMC Musculoskelet Disord. 2020 Mar 31;21:200. doi: 10.1186/s12891-020-03228-y (PMC7110672; doi:10.1186/s12891-020-03228-y)
Supplement: Supplementary file 1 — Additional file 1. Sex-specific classification of specific muscle strength, specific muscle power, and muscle quality of individuals with obesity compared to healthy individuals. [file 12891_2020_3228_MOESM1_ESM.docx]

**Additional file 1.** Sex-specific classification of specific muscle strength, specific muscle power, and muscle quality of individuals with obesity compared to healthy individuals.

|  | All subjects | | | Young adults/adults | | | Middle-aged/older adults | | |
| --- | --- | --- | --- | --- | --- | --- | --- | --- | --- |
| Classification | Men  (n=100) | Women  (n=103) | p-value | Men (n=51) | Women (n=41) | p-value | Men (n=49) | Women (n=62) | p-value |
| Specific strength | | | | | | | | | |
| Normal | 8 (8%) | 0 (0%) | **<0.001** | 5 (10%) | 0 (0%) | **0.019** | 3 (6%) | 0 (0%) | **0.013** |
| Low | 18 (18%) | 6 (6%) |  | 13 (25%) | 5 (12%) |  | 5 (10%) | 1 (2%) |  |
| Poor | 74 (74%) | 97 (94%) |  | 33 (65%) | 36 (88%) |  | 41 (84%) | 61 (98%) |  |
| Specific power | | | | | | | | | |
| Normal | 97 (97%) | 94 (91%) | 0.251 | 50 (98%) | 40 (97.5%) | 1.000 | 46 (94%) | 54 (87%) | 0.186 |
| Low | 2 (2%) | 7 (7%) |  | 1 (2%) | 1 (2.5%) |  | 1 (2%) | 6 (10%) |  |
| Poor | 1 (1%) | 2 (2%) |  | 0 (0%) | 0 (0%) |  | 1 (2%) | 2 (3%) |  |
| Muscle quality | | | | | | | | | |
| Normal | 26 (26%) | 6 (6%) | **<0.001** | 18 (35%) | 5 (12%) | **0.011** | 8 (16%) | 1 (2%) | **0.010** |
| Low | 0 (0%) | 0 (0%) |  | 0 (0%) | 0 (0%) |  | 0 (0%) | 0 (0%) |  |
| Poor | 74 (74%) | 97 (94%) |  | 33 (65%) | 36 (88%) |  | 41 (84%) | 61 (98%) |  |

Data are shown as number of participants and percentage. The p-value corresponds to the comparison between sexes.
